# Experiential contributions to social dominance in a rat model of fragile-X syndrome

**DOI:** 10.1098/rspb.2018.0294

**Published:** 2018-06-13

**Authors:** K. Saxena, J. Webster, A. Hallas-Potts, R. Mackenzie, P. A. Spooner, D. Thomson, P. Kind, S. Chatterji, R. G. M. Morris

**Affiliations:** 1 Simons Initiative for the Developing Brain, Edinburgh Neuroscience, 1 George Square, Edinburgh EH8 9JZ, UK; 2 The Patrick Wild Centre, Edinburgh Neuroscience, 1 George Square, Edinburgh EH8 9JZ, UK; 3 Centre for Discovery Brain Sciences, Edinburgh Neuroscience, 1 George Square, Edinburgh EH8 9JZ, UK; 4 Centre for Cognitive and Neural Systems, Edinburgh Neuroscience, 1 George Square, Edinburgh EH8 9JZ, UK; 5 Centre for Brain Development and Repair, Institute for Stem Cell Biology and Regenerative Medicine, Bangalore, 560065, India; 6 National Centre for Biological Sciences, Bangalore, 560065, India

**Keywords:** fragile-X, social dominance, hierarchy, *Fmr1*, autism-spectrum disorders, gene–environment interactions

## Abstract

Social withdrawal is one phenotypic feature of the monogenic neurodevelopmental disorder fragile-X. Using a ‘knockout' rat model of fragile-X, we examined whether deletion of the *Fmr1* gene that causes this condition would affect the ability to form and express a social hierarchy as measured in a tube test. Male fragile-X ‘knockout' rats living together could successfully form a social dominance hierarchy, but were significantly subordinate to wild-type animals in mixed group cages. Over 10 days of repeated testing, the fragile-X mutant rats gradually showed greater variance and instability of rank during their tube-test encounters. This affected the outcome of future encounters with stranger animals from other cages, with the initial phenotype of wild-type dominance lost to a more complex picture that reflected, regardless of genotype, the prior experience of winning or losing. Our findings offer a novel insight into the complex dynamics of social interactions between laboratory living groups of fragile-X and wild-type rats. Even though this is a monogenic condition, experience has an impact upon future interactions with other animals. Gene/environment interactions should therefore be considered in the development of therapeutics.

## 1. Introduction

The aim of this study was to investigate whether deletion of the fragile-X mental retardation protein (FMRP) would impact the expression of a social hierarchy in group-living laboratory rats. Fragile-X typically arises via CGG trinucleotide repeats in its promotor region of *Fmr1*, the X-linked gene that encodes FMRP and a leading monogenic cause of inherited learning disability and autism [1,2]. Heterogeneous clinical symptoms are observed, including macro-orchidism (enlarged testes), abnormal facial features and a high prevalence of epilepsy; other phenotypic features can include alterations in aggressive behaviour, attentional and cognitive deficits, and severe mental retardation [3,4]. Social withdrawal is also a prominent characteristic. New treatments are urgently sought for this autism-spectrum disorder.

Animal models of fragile-X began with studies using mutant mice [5] and only more recently turned to the use of rats. One focus has been on the hypothesis that deletion of *Fmr1* affects signalling downstream of metabotropic glutamate receptors (mGluRs). Specifically, synaptic changes have been observed in mice that mimic the human syndrome, including alterations in dendritic spines [6], an exaggeration of the capacity to induce or sustain activity-dependent long-term depression of synaptic efficacy in the hippocampus [7,8] and alterations in long-term potentiation in the amygdala [9,10]. Behavioural changes are seen when the mice are tested singly, including deficits in ‘flexibility’ such as repetitive behaviours [11,12], a reversal deficit in the watermaze [5], and reactivity to novelty when objects are experienced in changed places and testing contexts [13]. These behavioural changes may be linked to the underlying changes in activity-dependent synaptic plasticity that mediates the capacity to learn, remember and update stored information in response to change.

Some animal studies have also directly examined the social phenotypes of human fragile-X such as social withdrawal, social anxiety and difficulty in forming peer relationships. Impairments in social communication and social interactions have been reported [12,14–17], reflecting construct validity, as well as rodent-characteristic social behaviour such as ultrasound communication [18]. However, a key issue in examining any aspect of social behaviour is establishing the relative influence of genetic and environmental factors in the determination of phenotype because social interactions offer the opportunity for transitivity of dominance relationships and stability or change over time [12]. It is known that a rat's age is a significant correlate of success in dominance interactions by virtue of an animal's memory of previous social interactions with another animal [19]. This memory may be the basis of both ‘transitivity' (A > B, B > C and A > C) and the stability of such inter-animal dominance relationships.

Inspired by a prominent study of causal mechanisms contributing to social dominance in mice by [20], we chose to study ‘social dominance' and ‘social hierarchy' as relatively tractable indices of social interactions using the ‘tube test' [21]. We sought to contrast *direct* effects of the FMRP mutation on the expression of a social hierarchy, with *indirect* effects mediated by an individual animal's experience of its rank within its social group across repeated testing sessions. The simplest question is: does a cage of male *Fmr1^−/y^* rats living together develop a social hierarchy? Second, would wild-type rats be dominant in a social group consisting of both lines living together with *Fmr1^−/y^* rats? Third, beyond measurement of an individual's ‘rank' in such hierarchies, would *Fmr1^−/y^* rats show differential variability in rank over time? That is, might normal rats display stability by virtue of remembering previous encounters, but *Fmr1^−/y^* rats display a more variable pattern? Fourth, what would be the impact of an individual animal's experience of his rank upon his subsequent social interactions with a stranger animal (i.e. from a different cage)? That is, what would be the impact on future contests with strangers of a rat being highly or lowly ranked in his home cage? Would genotype or relative rank in the home cage dominate in determining the outcome? Pertinent to these questions is that a new study in mice suggests that synaptic plasticity on a thalamic–prefrontal pathway in the brain of mice, reflecting the history of winning or losing social contests, can be causally involved in determining an animal's rank [22].

We tested a new line of *Fmr1* knockout (KO) rats on a Long-Evans background originally developed in conjunction with Sage/Horizon (LEH rats [23]). This line was made using Zinc finger nuclease (ZFN)-mediated disruption of *Fmr1*. In a first report, Asiminas *et al.* [24] described in detail (1) the target site for ZFN cleavage, (2) the donor sequence, including eGFP and a nuclear localization signal flanked by 5′ and 3′ homology recombination arms for homology directed repair, and (3) the resulting targeted locus. Immunohistochemical localization of FMRP in P15 WT and Fmr1 KO rats shows the absence of FMRP in transgenic rats. This line has been calibrated by our colleagues as showing several of the same aspects of phenotype as previously observed in a different line of rat [13]. The switch from mouse to the rat is because rats display complex patterns of social behaviour that have been extensively described [25]. Our hope was that, by using this more social species, we might thereby create an alternative and potentially useful rodent model of the social anxiety phenotype of fragile-X humans.

## 2. Material and methods

### (a) Subjects

The subjects were adult male Long-Evans hooded rats (*n* = 56), weighing between 320 and 680 g across the three separate cohorts (two tested at 3 months, one at 6 months). Rats of similar weights were group housed, four rats in a cage since weaning, with *ad libitum* access to food and water. A 12 h light and dark cycle was maintained, with all the experiments carried out in the light phase between 9.00 and 17.00. *Fmr1^−/y^* male and wild-type littermate control rats were generated by mating female *Fmr1* heterozygotes crossed to wild-type Long-Evans hooded rats (Charles River Labs). The wild-type (WT) rats were littermate matched.

The experiment was conducted in three successive cohorts over 2 years by K.S., J.W., R.M. and A.H.-P. In each cohort, the rats were housed in three major groups: WT only, KO only and mixed WT and KO. In each case, there were four rats together in a cage, with the mixed group composed of two WT and two KO animals. In total, there were 56 rats divided between three WT cages, four KO cages and seven mixed cages. In all the cases, the experimenters were blind to the genotype of the subjects, the animals in each cage being colour-coded on their tails, with the code made and retained by independent person (DT).

### (b) Apparatus

**Figure 1.**
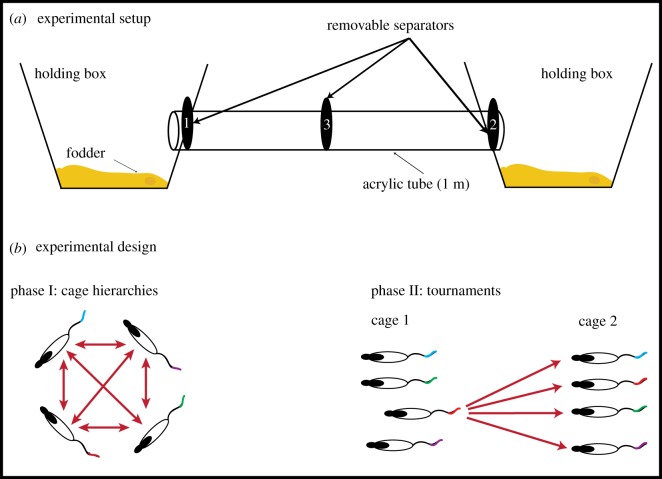
Experimental apparatus and design. (*a*) An acrylic (Perspex) tube 1 m in length connected two holding boxes containing ‘bedding' from the home cages of the animals being tested. Three separators limited access to the tube from the cages (1 and 2), and at the centre (3), contact between the two animals. A trial commenced when the central separator was lifted. (*b*) The design involved two phases. Phase I involved measurement of the relative rank and variability in rank within each of the cages. Phase II involved competitions between each animal of one cage against each animal of another cage (e.g. rat 3 of cage 1 against each of the four animals of cage 2). Note colour coding of the animals with a tail mark.

The tube test was adapted for rats from the study of Wang *et al.* [20]. A transparent plastic tube 1 m in length (small tube: 6 cm internal diameter; large tube: 7 cm internal diameter) was placed between two holding boxes (42 × 26 × 18 cm; figure 1*a*). The tube size was selected so that rats could easily run down the tube, but they were unable to pass each other. Removable barriers were placed at the entrances of the tube from each holding box, and at a central location. Bedding from the rats' home cage was placed in each holding box. The tube and boxes were placed on a table in a laboratory room with subdued lighting, the experimenter at a table facing the equipment. A video system was used to record selected trials for illustrative purposes only.

### (c) Protocols

#### (i) Habituation

Each cage was handled daily for a minimum of 3 days prior to testing. During a single day of habituation, all animals in a cage were placed singly in the apparatus and allowed to run freely in the tube between the holding boxes for 30 min. All barriers were removed. Small sections of tubing had also been placed in all home cages 2 weeks prior to testing to help familiarize the animals.

#### (ii) The tube test of social dominance

Two rats were tested by placing one rat at either end of the tube in their respective holding boxes. The entrance barriers were then lifted and the rats introduced into the tube, whereupon they generally moved to the centre. Once both rats touched the central barrier, it was raised to allow the dominance test to begin. The rats competed for dominance in the tube in various ways, ranging from submission and retreat by one of the animals through to direct physical pushing of one opponent backwards out of the tube. ‘Winning’ was defined as reaching the entrance barrier slit at either end of the tube. There was never any ambiguity about which rat had ‘won'. Each pair of rats was run five times, with the winning rat recorded each time and the side on which each rat started swapped between successive runs (to counterbalance left and right). The time between lifting the middle barrier and the submissive animal leaving the tube was recorded as the latency (s). The apparatus was cleaned with an amphoteric disinfectant between cages but not between all encounters. Cage order was varied so that start times per cage changed between trials. A random order generator assigned pair orders within cages, which changed between sessions. In each session, fodder from the home cage was placed in the holding boxes to mirror the home-cage environment.

#### (iii) Phase I: dominance hierarchies

The tube test was initially used to establish within-cage dominance hierarchies (figure 1*b*, phase I). In each session, every animal competed against all three of its cage-mates. Each pair was run in the tube five consecutive times. A rat winning at least three times in a five-trial block was declared a clear winner, but testing always continued for five trials. A winning rat was allocated two points and the loser rat given 0 points per trial. Tallying up these points across all five trials allowed dominance ranking of the four cage-mate rats. In the case of equal points, the ranking was decided based on the outcome of the specific competition between the two equal scoring individuals, with the winner ranked higher. This test of position in the hierarchy was repeated 10 times across successive sessions.

#### (iv) Phase II: inter-cage tournaments

The second phase was to assess dominance in novel social conflicts (figure 1*b*, phase II). Because the full experiment was conducted as three separate cohorts, we could only test a subset of the inter-cage competitions. Four combinations were tested as follows: WT versus KO (two cages), mixed versus mixed (three cages), WT versus mixed (two cages), and KO versus mixed (two cages). Each rat competed against every animal from the paired cage (total 16 pairs), with five runs per pair per day. A random order generator established pair order as well as cage order. Each tournament was repeated three times across days. The tube was changed between pairs and fresh bedding was laid down in the holding cages for each tournament. Before testing, one cage was allowed to freely explore the tube for 5 min, followed by the second for 5 min and the first again for 2 min, ensuring no particular animals/cages smell dominated the environment. The cages were renumbered by a third party to keep the experimenter blind.

### (d) Statistical analyses

Test for the statistical significance were used. A *χ*^2^-test was used to compare the expected and observed values of number of wins between genotypes. A *t*-test was used to compare average rank and wins per pair between WT and KO in mixed cages. Where the statistical variance of rank and/or a simple change/no-change measure of rank stability is presented, a *t*-test was used to test the level of significance. For the variance scores, we adjusted the values to a percentage score in which 100% was the maximal variance score possible. Owing to the presence of two different genotypes in the mixed cages, their analysis was processed separately to that of the single-line WT and KO cages. The Pearson correlation coefficient was also computed to examine stability across sessions. The significance level is quoted for all tests. All the plots are represented as mean ± s.e.m.

## 3. Results

### (a) Qualitative observations

The rats behaved appropriately in the tube test, readily walking down the tube to the centre, confronting their ‘opponent', investigating him and interacting socially. Upon opening the partition separating the two sides, the two animals investigated each other and then either advanced or retreated as appropriate (figure 1). Very little aggressive behaviour was observed. Latencies to complete individual tests typically ranged from 3 to 30 s (rarely longer), with considerable within and between animal variability (such that latency did not prove to be a useful measure). As described in the Material and methods, the contests between two individuals were repeated five times each day to ascertain a clear ‘winner' of each session.

### (b) Phase I: dominance hierarchies

**Figure 2.**
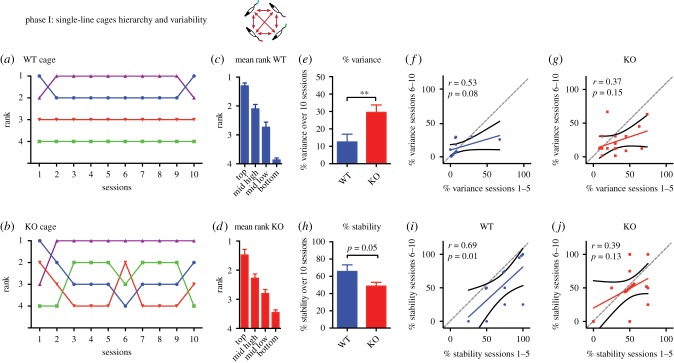
Cage hierarchies—single-line cages. (*a,b*) Representative session rankings of individual colour tail-marked rats in a single-line WT cage (blue) and a single-line KO cage (red) across 10 days of testing. Note greater instability of rank of the KO rats. (*c,d*) Mean rank of rats as a function of their ordinal position in a cage hierarchy averaged across 10 days. Note clear hierarchy of WT single line (three cages) and KO single line (four cages). (*e*–*g*) Normalized percentage variance of rank for WT and KO rats across 10 sessions of testing, and correlation between variance observed during sessions 1–5 and 6–10 for WT and KO rats respectively. (*h*–*j*) Normalized percentage stability for WT and KO rats across the same sessions as (*e*–*g*). **p < 0.01; mean ± 1 s.e.m.

The first result, secured over 10 successive sessions of testing, was that a clear social hierarchy was apparent in both the single-line cage groups containing only WT or only *Fmr1^−/y^* KO animals (figure 2). Figure 2*a*,*b* show the stable and shifting rank of animals in two representative single-line cages over the full 10 days of testing; figure 2*a* displays an example of a WT cage that was particularly stable, whereas figure 2*b* shows a KO cage displaying apparently greater day-to-day variability of rank. From the full set of seven single-line cages, the average rank over 10 days collectively revealed a clear hierarchy in both lines; the data is plotted to show the highest (top), lowest (bottom) and the two mid-ranked animals across cages (figure 2*c*,*d*).

Second, we then sought to measure the apparent variability in rank across sessions. Two separate measures were used: (a) *variance*—the statistical measure of variability of absolute rank across sessions (normalized in each animal to 100% for a maximum possible variance of 2.5 across 10 sessions, and then averaged); (b) *stability*—measured as the count of the number of times an animal retained its rank from one session to the next, without regard to extent of the change (normalized in each animal to a score of 9 equalling 100% and then averaged; based upon the measure used by the Hu laboratory [20]). Figure 2*e* shows that percentage variance was significantly lower in the WT single-line cages than in the KO cages (*t* = 2.99, d.f.= 26, *p* = 0.006). Stability showed a similar trend (figure 2*h*), although the change did not reach statistical significance (*t* = 2.05, d.f. = 26, *p =* 0.054). We then examined whether variance and stability changed across the 10 sessions. With each measure calculated separately for sessions 1–5 and 6–10 (figure 2*f*,*g*,*i*,*j*), despite 5 of the 12 WT rats showing a decline in stability across sessions, this group showed a significant correlation of rank stability across the two subsets of five sessions (single-line cages, *r* = 0.69, *p* = 0.01). By contrast, the KO rats did not show a significant correlation of rank stability across sessions (*r* = 0.39, *p* = 0.13). The corresponding data for variance are difficult to interpret as the ‘clumping’ of the very low variance scores for the WT rats differs from the much more dispersed scores of the KO rats (figure 2*f*,*g*). In both cases, the trend was for the slope of the regression line to be lower than 45°, pointing to a gradual increase of instability across repeated tube-test sessions.

**Figure 3.**
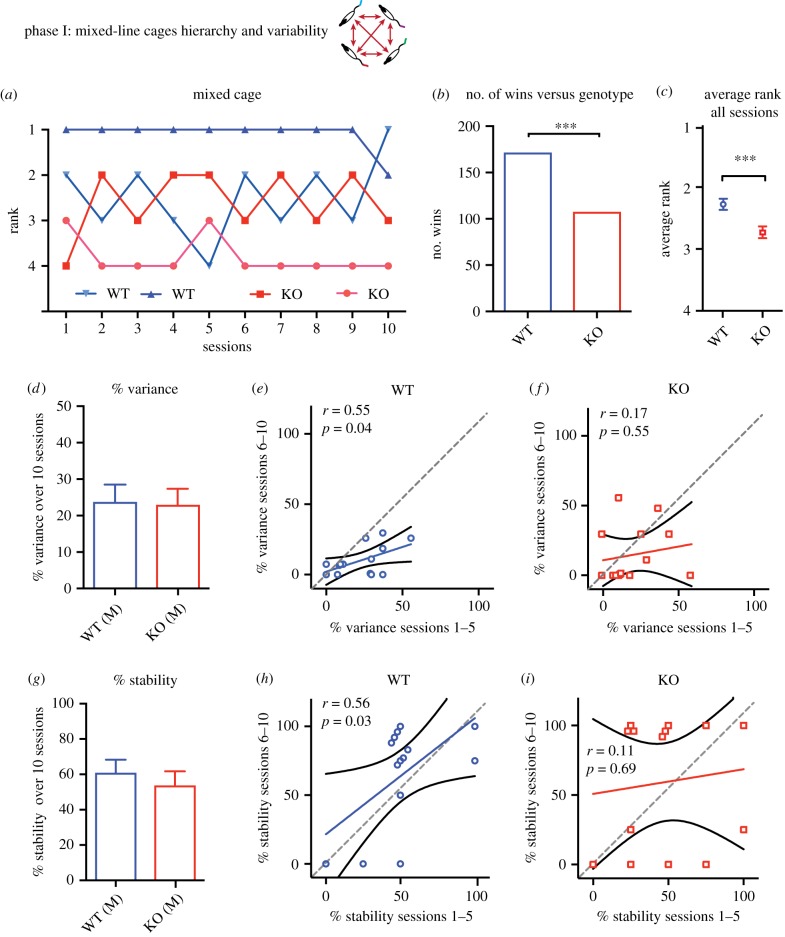
Cage hierarchies—mixed-line cages. (*a*) Session rankings of individual colour tail-marked rats in a representative mixed-line cage across 10 days of testing (colour-coding as in figure 2). Note relative but not absolute dominance of WT rats. (*b,c*) Number of wins of tube-test contests by genotype and the distinct but non-independent measure of mean rank. See text for *χ*^2^ statistics. (*d*–*f*) Mean percentage variance of rank for WT and KO rats across 10 sessions of testing, and correlation between variance observed during sessions 1–5 and 6–10 for WT and KO rats, respectively. (*g*–*i*) Mean stability for WT and KO rats across the same sessions as (*d*–*f*). Note that WT rats show inter-session predictability between sessions 1–5 and sessions 6–10, whereas KO rats in these mixed cages divide bi-modally into two sub-groups with low and high stability over sessions 6–10. ***p < 0.001; mean ± 1 s.e.m.

We then, third, examined the mixed-line cages in each of which two WT and 2 KO rats had been living together for several weeks. The key finding was that WT rats won significantly more contests than KO animals (figure 3). This is shown for a representative cage (figure 3*a*), in the analyses of the overall number of contests (*χ*^2^ = 14.63, d.f. = 1, *p* = 0.0001; figure 3*b*), and for the average rank of individual WT (2.27) and KO (2.72) animals calculated across all 280 contests (*t* = 3.48, d.f. = 278, *p* = 0.0006; figure 3*c*). We wondered whether there was any relationship between rank and weight of the animal, surmising that larger animals might be more inclined to win. The concern was that, in comparing WTs and KOs in animals living together, the comparison of genotypes might be secondary to a difference in weight. However, not only was there no relationship between weight and rank (*r* = 0.057, NS; data not shown), the mean weight of the WT rats (468.4 g) was, if anything, slightly lower than that of the KO animals (488.8 g; NS). Thus, the apparent dominance of the WT over the KO animals is not an artefact of greater weight.

As in the single-line cages, both variance and stability were computed across the 10 sessions, and separately for sessions 1–5 and 6–10. No overall mean differences were observed (figure 3*d*,*g*). However, in examining change across the two subsets of five sessions, only the WT animals displayed significant correlations of variance (*r* = 0.55, *p* = 0.04) and stability (*r* = 0.56, *p* = 0.03). The KO rats showed non-significant regression coefficients (*r* = 0.17 and *r* = 0.11, N.S.), reflecting instability of rank, but interestingly, with correlation slopes that were well below 45°. Strikingly, the KO rats subdivided across sessions into ‘hyper-stable' (*n* = 8) and ‘hyper-unstable' (*n* = 6) sub-groups (note that such stability and instability may reflect either a high or low absolute rank, figure 3*i*).

Thus, KO rats have a phenotype reflecting lower dominance than cage-mate WT rats, reflecting social withdrawal, but apparently greater variability in their experience of tube-test contests that may affect their future interactions across sessions.

### (c) Phase II: inter-cage tournaments

**Figure 4.**
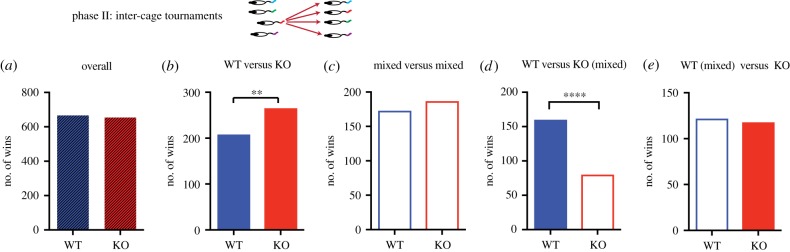
Cage tournaments—organized by genotype*.* (*a*) When all WT and KO rats from different cages met in contests in each of the three cohorts (resulting in 1320 contests), there was a loss of the phenotype that in phase I had reflected dominance by WT rats. Hatched colour coding reflects pooled data from both single-line and mixed-line cages. (*b*) Significant dominance of KO rats in single-line cages in contests with equivalent single-line WT rats. (*c*) No dominance pattern when the mixed-line cage animals competed against each other. (*d*) WT rats from single-line cages were dominant over KO mixed-line rats. (*e*) No dominance pattern when the WT mixed-line cage animals competed against KO single-line rats. ****p < 0.0001; **p < 0.01; mean ± 1 s.e.m.

The second phase of experimentation involved ‘tournaments' in which all animals of a given cage were, over three sessions, pitted against all animals of another cage, with five trials per pair per session (figure 4). These contests were therefore always with ‘stranger' animals (note that this could not be done for all 14 cages of phase I as the study was conducted in three replications; during any one replication, the subset of cages that could be tested was against others of that same replication). There were, nonetheless, a total of 1320 contests (sufficient for statistical reliability). This phase allowed us to ask, over all permutations, whether the dominance rank an animal had assumed by the end of phase I was predictive of his behaviour in the tournament tests of phase II.

Surprisingly, comparison of WT and KO rats now revealed no difference as a function of genotype (figure 4*a*; *χ*^2^ = 0.11, d.f. = 1, *p* = 0.74, NS); the phenotype displayed in phase I appeared to be lost. Comparison of the cage tournaments between only the single-line cages revealed, paradoxically, that KO rats won *more* contests against WT rats (*χ*^2^ = 7.00, d.f. = 1, *p* = 0.0081; figure 4*b*). The situation for the mixed cages, for which the KO rats would have had more experience of losing than WT rats, was more evenly matched (*χ*^2^ = 0.54, d.f. = 1, *p* = 0.46, NS; figure 4*c*). In keeping with an emerging pattern, when single-line WT rats were pitched against mixed-cage KO rats, there was a significant dominance of the WT animals (*χ*^2^ = 27, d.f. = 1, *p* < 0.0001; figure 4*d*). This might have been expected to continue for the comparison of WT rats of mixed-line cages versus KO single-line cages (figure 4*e*), but this was not observed—possibly because WT rats that had experience of KO rats were for some reason wary of stranger KO rats (*χ*^2^ = 0.06, d.f. = 1, *p* = 0.79; NS; figure 4*e*).

**Figure 5.**
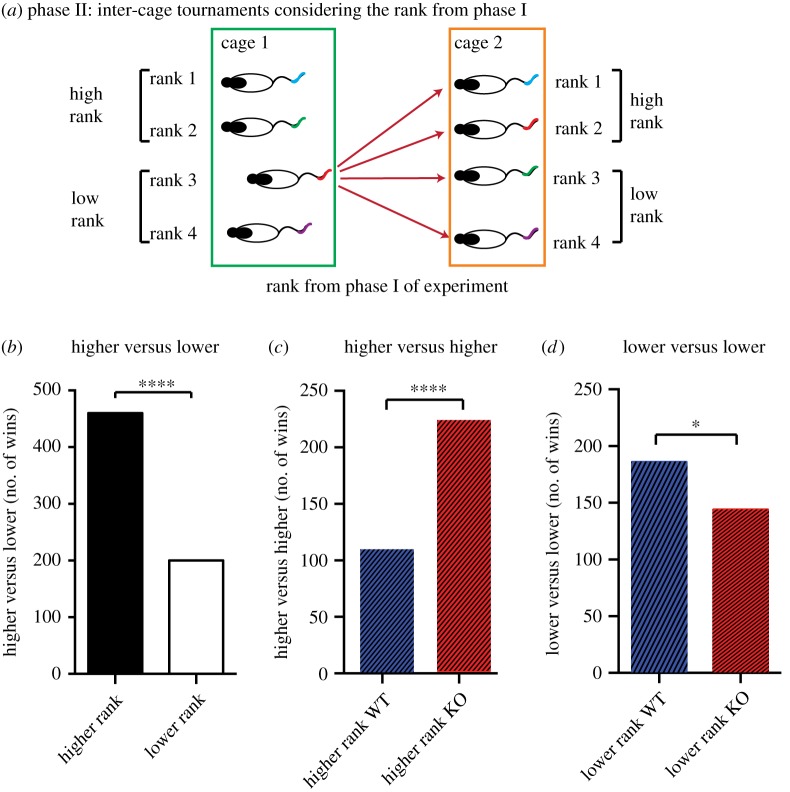
Cage tournaments—organized by experience of winning. (*a*) The data of figure 4 were re-examined taking into account the rank assumed in phase I of the study (contests with cage-mates). All pairwise contests between cages were tested (three times) and the cartoon depicts a rank 3 animal in cage 1 competing against all rats of cage 2. (*b*) Rats that had previously been higher ranked (ranks 1 and 2) were dominant over stranger rats that had been lower ranked (ranks 3 and 4), when scores were computed without regard to genotype. (*c,d*) Subsets of the cage tournament data analysed only for the contests between higher-ranked WT versus higher-ranked KO animals, and separately for low-ranked WT versus low-ranked KO animals. When previous ranking and genetic line were considered together, the dominance by KO rats in phase I was specific to the higher-ranked animals. For lower-ranked animals, WT rats were dominant were marginally but significantly dominant. ****p < 0.0001; *p < 0.05; mean ± 1 s.e.m.

The impact of experience gained from the contests of first phase was most striking when the same data of figure 4 were re-examined with regard to phase I rank as well as genetic status (figure 5). Figure 5*a* shows a cartoon of the rank (1–4) of the four rats in a given cage (cages 1 and 2) with each animal of cage 1 tested against each ‘stranger' animal of cage 2 (graphically representative example shown for the rank 3 animal of cage 1). The phase I rank was partially predictive of such contests because, when animals of both genotypes were pooled together regardless of genotype into a sub-group that had first or second rank in phase I (high ranked, figure 5*a*) and had gone through the contest with the sub-group of third or fourth ranked animals (low ranked) (i.e. excluding higher versus higher and lower versus lower), the higher-ranked individuals won significantly more contests (*χ*^2^ = 102, d.f. = 1, *p* < 0.0001; figure 4*b*). While such a result may seem trivial, this suggests that the experience of winning or losing over the 10 days of testing in phase I has consequences for future interactions with the strangers.

It then became of interest to ask the outcome with respect to genetic status of stranger-contests between higher-ranked and separately between lower-ranked animals. Strikingly, KO high-rank rats won significantly more often against WT high-rank animals (*χ*^2^ = 39, d.f.= 1, *p* = 0.0001; figure 5*c*), but this pattern was reversed in the comparison of the WT and KO low-ranked animals (*χ*^2^ = 5.3, d.f.= 1, *p* = 0.02; figure 5*d*). Note the number of contests in figure 5*c*,*d* are necessarily half those of figure 5*b*, which is half of overall contest number of 1320 (figure 4*a*) as it (figure 5*b*) excludes the competition between the higher versus higher and lower versus lower.

## 4. Discussion

Previous studies of social dominance in animal models of autism-spectrum disorders have focused on mice due to the relative ease of making deletions of murine genes by homologous recombination [12,26]. One novel feature of this study is the use of the rat following the development of this ‘knock-out' rat-model of fragile-X syndrome [24]. Accordingly, we sought to examine whether deletion of FMRP would have an impact on (a) the ability to form and express a social hierarchy as measured in a tube test, (b) social dominance behaviour when animals are confronted by another rat living in the same cage and (c) social dominance when confronted by a stranger rat from another cage.

### (a) The tube-test measure of hierarchy

Our data indicate that male *Fmr1^−/y^* rats living together in cage groups of four animals readily form a social hierarchy that can be measured reliably using the tube test (which is known to correlate well with several other measures of social dominance in mice [20]. However, in groups of animals consisting of 2 WT and 2 *Fmr1^−/y^* rats, the WT rats were significantly more dominant, although not exclusively so on a rat-by-rat basis. Thus, there is a clear social dominance phenotype associated with this rat model of fragile-X that may correspond to aspects of the social anxiety and/or social withdrawal of affected individuals when confronted by others.

A single tube test encounter has only one output—one animal is a winner (dominant) and the other a loser (subordinate). By testing every member of a cage with all others, and doing so several times within a session (to ensure within-day reproducibility), we could decipher at least one measure of the social hierarchy in that cage and how it evolved over successive days of testing. Other tests examined by Wang *et al*. [20] include whisker trimming, also known as the ‘Dalila effect', but this phenomenon is observed in mice and not in rats [27]. A urine marking assay, in which the so-called dominant animals are observed to mark a larger territory than subordinates, was a further possibility; however, one study observed that male rats spend more time adjacent to the urine of other male rats than to an odourless substance, pointing to a lack of avoidance of urine marking under some conditions [28]. It has also been observed that sniffing frequency decreases in the subordinate animal of a pair [29], and we could examine this in a follow-up study (see below). The extent of access to food or water in a group cage is potentially another measure, but was not suitable for us as it requires the food and/or water deprivation which we did not use; resource competition can, however, be well correlated with the tube test in rats [30]. As these measures, where they could be measured, correlated well for mice (see [18]), we believe that our focus on the tube test is justified.

### (b) Interaction of genetic and experiential factors

This study revealed the impact of both genetic and, to a limited extent, experiential factors. One aspect of the latter was repeated testing over several days, and this revealed greater dominance stability in WT rats than *Fmr1* KO animals. Moreover, the rank held by a WT rat during the first 5 days of testing was predictive of its rank during the second 5 days of testing in both single-line and mixed-line cages. This is not surprising as rats readily form a relatively stable hierarchy—in laboratory conditions [31] and in the (semi-)wild [19]. Stability is also promoted under laboratory conditions when group-living animals must compete for food resources from the time of weaning [32]. In contrast, *Fmr1* KO animals displayed changes in stability over the course of the 10 days of testing. The second experiential aspect we examined was the impact on social dominance of prior experience of testing with familiar home-cage animals upon later tournaments with stranger rats. This revealed an apparent loss of the earlier identified genetic phenotype, with social dominance behaviour now dominated by the animals' prior experience of having won or lost earlier contests. That is, rats of either genotype ranking relatively highly in phase I won more phase II contests against animals ranked relatively lowly (although again not exclusively). This is consistent with the memory of ‘previous outcome' concept with respect to social dominance [19]. However, when this expected pattern was broken down into sub-groups reflecting both genotype and relative rank, a more complex picture emerged that, if anything, constituted only a partial reversal of the earlier phenotype.

One intriguing possibility is that KO rats fail to process the social cues from the opposing animal as effectively as do WT rats. Thus, in considering the pattern shown during the later contests with stranger rats, we suspect that an important aspect of the animals' experience was not just rank but the relative variance or stability of the rank, with a phenotype clearly emerging to the effect that the WT rats showed greater stability (66%) than KO rats (49%). These absolute levels are commensurate with those reported for one study in mice that measured stability across testing sessions (59% stability [20]). However, as the testing sessions of phase I continued, the KO rats showed a bimodal pattern of becoming either ‘hyper-stable' or ‘hyper-unstable’. This pattern is reminiscent of the autism symptom of repetitive behaviour. As noted above, our observations and scoring of the animals were conducted blind to genotype, but we sensed that some animals behaved as though sensitive to cues from the other animal whereas other animals behaved as if they were not. If there is a genotype-associated difference in the processing of social cues, KO rats may develop the behavioural patterns of either pushing forward or resisting push behaviour from the other animal regardless (‘bully' strategy) or retreating the moment another animal is experienced (‘submissive' strategy). Both of these patterns would then populate the sub-group showing ‘hyper-stability' of high rank and low rank, respectively. If this analysis is correct, it would be expected that high-ranked KO rats displaying hyper-stability would win over stranger WT rats, even high-ranked WT rats, whereas low-ranked KO rats displaying hyper-stability would lose in contests with stranger WT rats, even low-ranked WT rats. This is likely to have contributed to the pattern that was observed. In future research, it would, therefore, be valuable to video carefully all encounters between animals in the tube test, as done with mice [22,33], with a view to objectively identifying patterns of behaviour that may reflect differential sensory processing (e.g. whisker movements), motor movements related to advance or retreat and social interactions (e.g. head contact, overt aggression). This would be demanding to do because it would probably require a replication and video capture of all conditions described here, again conducted and filmed ‘blind' over hundreds of encounters, but then decoded with respect to genetic status and previously established rank.

Studies of social dominance by Paylor's group [14] were the first to establish that *Fmr1* KO mice displayed abnormalities in the tube test. This pioneering work also showed that experience matters when studying encounters between familiar (cage-mate) and unfamiliar animals, and between mice that had experience of tube-test encounters compared with mice on the first day of testing. Specifically, they observed either WT dominance or equivalence of the two genotypes as a function of these experiential factors [14], a finding replicated by Goebel-Goody *et al*. [34]. More recently, however, a study by de Esch *et al.* [35] reported a variety of tube-test outcomes, as a function of differential housing and experience. Unlike our results from cage-mates during phase I, they observed greater dominance by *Fmr1* KO over WT mice that was highly significant. Dominance increased and stabilized over 5 days of testing, apparently the opposite of our findings with rats. De Esch *et al.* [35] looked at contests between WT and KO rats that were living in separate cages, as we did also in phase II, in which we also had a similar result of KO dominance over WT rats (figure 4*b*). However, we only observed this reversal from phase I for contests between single-line cages; we saw no more than a trend for this change in phenotype for mixed-line cages (figure 4*c*). De Esch *et al.* [35] went on to establish that knocking out the mGLUR5 sub-unit of metabotropic glutamate receptors in *Fmr1* KO mice had a dramatic effect. Specifically, *Fmr1* KO animals won tube-test encounters against animals with the double knockout, the double knockout animals won against WTs, but the WTs won against animals with only the mGLUR5 knockout. We are uncertain what to conclude from this pattern of results except to agree with Spencer *et al.*'s [14] assertion that in future investigations of *Fmr1* KO animals, it will be necessary ‘to incorporate experimental designs to clearly assess the influence of test experience on behaviour’ (p. 428). Whether the use of rats (as here) rather than mice will help in this task remains to be established, but a strong case has been made for their use now that technologies for rat genetic interventions are becoming available [36]. From the perspective of brain mechanisms, Zhou *et al.* [22] have recently provided that neural activation of the dorsomedial prefrontal cortex is both necessary and sufficient to induce dominance in tube-test social competitions. The causal evidence derives from both chemogenetic inhibition studies and optogenetic activation experiments directed at a thalamic–prefrontal pathway. In a further demonstration of the validity of measures from the tube test, transfer from it to a ‘warm-spot' test in a cage with an otherwise ice-cold floor was shown to display a significant correlation of these two measures of rank. They note that ‘an important parameter for the cost-benefit computation in a social confrontation is the history of winning … our results might shed light on the treatment of … psychiatric diseases’ ([22], p. 168).

There is growing interest in the interaction of genetic and environmental influences on the cognitive phenotype of fragile-X children [37], and wider interest in psychiatry [17]. It would seem to be of value to continue our analysis of gene–environment interactions in this rat model of fragile-X, diversifying into a wider set of tests of social interaction followed by exploring the impact of pharmacological treatments that target abnormalities in mGLUR signalling.

## Data accessibility

Data presented in this article are available to the public via https://datadryad.org and can be accessed with the Dryad Digital Repository (http://dx.doi.org/10.5061/dryad.mh352th) [38].
